# Genome-Wide Identification and Expression Profiling of Pathogenesis-Related Protein 1 (*PR-1*) Genes in Durum Wheat (*Triticum durum* Desf.)

**DOI:** 10.3390/plants12101998

**Published:** 2023-05-16

**Authors:** Ikram Zribi, Mouna Ghorbel, Najla Haddaji, Malek Besbes, Faiçal Brini

**Affiliations:** 1 Laboratory of Biotechnology and Plant Improvement, Centre of Biotechnology of Sfax, P.O. Box 1177, Sfax 3018, Tunisia; ikram.zribi@cbs.rnrt.tn; 2 Department of Biology, College of Sciences, University of Hail, P.O. Box 2440, Ha’il City 81451, Saudi Arabia; m.ghorbel@uoh.edu.sa (M.G.); n.haddaji@uoh.edu.sa (N.H.); m.besbes@uoh.edu.sa (M.B.)

**Keywords:** abiotic stress, durum wheat, gene expression, pathogenesis-related proteins, in silico analysis

## Abstract

Pathogen-related proteins (PRs) are diversified proteins with a low molecular weight implicated in plant response to biotic and abiotic stress as well in regulating different functions in plant maturation. Interestingly, no systematical study has been conducted in durum wheat (*Triticum turgidum* subsp. *durum*). In the present study, *12 PR-1 genes* encoding a CAP superfamily domain were identified in the genome of *Triticum turgidum* subsp. *durum*, which is an important cereal, using in silico approaches. Additionally, phylogenetic analysis showed that the *PR-1* genes were classified into three groups based on their isoelectric point and the conserved motif domain. Moreover, our analysis showed that most of the TdPR-1 proteins presented an N-terminal signal peptide. Expression patterns analysis showed that the *PR-1* gene family presented temporal and spatial specificity and was induced by different abiotic stresses. This is the first report describing the genome-scale analysis of the durum wheat *PR-1* gene family, and these data will help further study the roles of *PR-1* genes during stress responses, leading to crop improvement.

## 1. Introduction

Plants grow under constantly varying biotic and abiotic (salty soils, drought, waterlogging, UV lights, extreme temperatures, heavy metals, and many others) stress conditions. These factors are detrimental to plant maturation as they cause many metabolic perturbances and are the main cause of cell death [1,2]. To overcome such constraints, plants have evolved different mechanisms such as the accumulation of different phytohormones (abscisic acid (ABA), ethylene (ET), jasmonic acid (JA), methyl jasmonate (MeJA) and salicylic acid (SA), phytoalexins, and different defense genes (antioxidants, Mitogen Activatd proteins Kinase). These proteins control the accumulation of small proteins known as Pathogen-related proteins (PR proteins) which inhibit pathogen multiplication in uninfected plant organs [3].

PRs are a group of proteins presenting low molecular weight that accumulate in plants during plant maturation, gene expression, flowering, plasmolysis, and senescence [4,5]. Moreover, several studies have shown that PR proteins are accumulated following pathogen infection. They control plant production of different peptides, proteins, or compounds which prevent pathogen infections where they start or act as toxins to pathogens [6]. As a result, plants increase their tolerance to protect themselves against pathogens [5]. In addition, PR proteins respond to various types of abiotic stresses such as drought, freezing, UV-lights, salinity, light, and osmotic stress [7,8] and hormones such as salicylic acid (SA) [9], abscissic acid (ABA), jasmonic acid (JA), and auxin (IAA) [8,10,11]. The PR proteins are thermostable, protease-resistant proteins that have a molecular weight of ~5–43 kDa. In addition, the *PR*-genes are expressed in all plant organs. Interestingly, they constitute about 5–10% of the total protein in leaves [12]. PR proteins have been classified into 19 different families based on their main properties such as their protein sequence similarities, enzymatic activities [13], pH (acidic or alkaline), antigen 5, cysteine-rich secretory proteins, and pathogenesis-related-1 (CAP) proteins [14]. Among the different PR classes, PR-1 proteins were the first discovered proteins and the most studied. In 1970, PR-1 proteins were identified in *Nicotiana tabacum* plants infected with Tobacco Mosaic Virus (TMV) [15]. Subsequently, different PR proteins, especially PR-1 proteins, have been isolated in many plant species. In fact, 32 and 22 *PR-1* genes were identified in rice and *Arabidopsis thaliana*, respectively [16], 17 in tea [17], 23 in wheat [18], 11 in black pepper [19], 24 in soybean [20], 21 in grape [21], 18 and 14 in cassava and black cottonwood, respectively [22], 19 in sugarcane [23], 13 in tomato [7], and 15 and 11 in banana (A and B genomes, respectively) [24].

PR-1 are ubiquitous proteins used as markers for systemic acquired resistance (SAR) by inducing programmed cell death. Thus, they ensure the reduction of pathogen growth [25,26]. Moreover, those proteins have anti-fungal, anti-bacterial, anti-viral, anti-insecticidal, and anti-nematode activities [5,9]. Cysteine-rich secretory protein, antigen 5, and pathogenesis-related 1 (CAP) are the conserved domains characteristic of all PR-1 proteins [4]. The CAP domain is known by its antimicrobial activity due to the caveolin-binding motif. Thus, it is responsible for the binding of sterols present in the membrane of the pathogen [27,28]. Structurally, the CAP domain conserved structure folds into four α-helices and one–four strands of beta-sheets, stabilized by disulfide bridges. Similar structure characteristics were shown overall in PR-1 proteins [16,29,30]. Such unique structures are indispensable for their biological roles in response to different biotic and abiotic stresses [31]. In fact, in sugarcane, 19 different PR-1 proteins were identified that respond to a wide range of stresses such as infection with *Acidovorax avenae* subsp. *avenae* (*Aaa*), as well as other abiotic stresses such as NaCl, PEG6000, and SA treatments [23]. In *Arabidopsis*, it has been shown that an ELF18-INDUCED LONG NONCODING RNA 1 (ELENA1) acts as a positive regulator of immune responsive genes during their transcription [32]. Moreover, ELENA1 associate with a Mediator subunit 19a (MED19a) to enhance the enrichment of the complex on the *PATHOGENESIS-RELATED GENE 1* (*PR-1*) promoter, whereas FIBRILLARIN 2 (FIB2) are negative transcriptional regulators of different immune responsive genes, such as *PR-1*. ELENA1 can dissociate the FIB2/MED19a complex. Thus, it releases a FIB2 transcriptional regulator from the *PR-1* promoter and enhances PR-1 expression [32]. SlPR-1, a tomato PR-1 protein, was induced after plant treatment with SA and infection with *Meloidogyne incognita* nematode [7], whereas PR-1b1 was induced after plant exposure to chilling temperatures [33]. An accurate tolerance to fungi and bacteria has been proved by over-expression of PR-1 in transgenic plants [26,34,35]. In the bread wheat, *TaPR-1-1* gene expression was induced by osmotic stresses, freezing, and salinity. Interestingly, the overexpression of TaPR1-1 positively regulated plant tolerance to those stresses in yeast and *Arabidopsis* [36]. In oat (*Avena sativa* L.), AvPR-1 was induced after plant exposure to different phytohormone applications and various abiotic stresses such as NaCl, mannitol, PEG, and heat [10]. Interestingly, and despite the extensive work on PR-1 proteins, little is known about their regulation. In *Arabidopsis*, it has been demonstrated that PR-1 protein interacts with AtWRKY50, which is considered the most effective WRKY activator of *PR-1* gene expression [37]. In fact, AtWRKY50 interacts with the *PR-1* promoter via its C-terminal portion. This interaction occurs simultaneously in the presence of TGA2 or TGA5 and AtWRKY stimulates this binding [37]. The same result was also found in tobacco. In fact, NtWRKY12 and TGA2.2 interact in the regulation of tobacco *PR-1a* promoter activity [38].

The tetraploid wheat (*Triticum turgidum* subsp. *durum* (*Desf.*) Husn. is an important staple crop for food security around the world [39].

About ten thousand years ago, the origin of durum wheat cultivation was concentrated in the fertile crescent [40]. Durum wheat was almost the principle constituent of both popular local dishes such as bulgur, couscous, semolina, frike, pasta, and bread wheat flour, and national identities [41].

Since then, durum wheat cropping has constituted an important industrial and economical crop in those regions. For instance, North African countries are considered among the largest exporters of pasta to Sub-Saharian Africa by using 100% durum semolina to respond to the international standard of ‘pasta’. Its high production and quality are linked to the adaptation and resistance of durum wheat to climatic stresses [39].

During human invasion, interesting durum wheat varieties (yielding, tasty, resistant to different environmental conditions) were domesticated and selected by farmers as well as by nature, which affected the genetic evolution by adapting to the environmental conditions in the Mediterranean areas [42]. Concerning the genetic structure of Tunisian durum wheat, it had been constructed not only by selection but also by the seed exchanges between farmers [41].

Indeed, Tunisian durum is known by its allelic richness and ability to resist drought [42] and to tolerate biotic stresses, such as the Fusarium head infection [43] and *Septoria tritici* blotch disease (STB), the causal agent of the most agronomically and economically destructive fungi, *Zymoseptoria tritici* [44]. The latest agent can cause a loss of yield with an average of 5–35% [45]. Durum wheat (Tunisian varieties) is also resistant to the wheat stem rust disease caused by *Puccinia graminis* f.sp. tritici [46] and to the cereal cyst nematode attack via the (TRITD2Av1G265560) gene, a potential candidate for improving wheat resistance to nematode infections [47].

After the green revolution, traditional varieties were abandoned and farms replaced these landraces with uniform modern cultivars; therefore, genetic erosion has occurred [48]. Recently, scientists have highlighted the problem of the unexplored Tunisian durum accessions to exploit the Tunisian genes’ quality and quantity [42,47]. Regarding its nutritive importance in comparison with common wheat, we investigated the genetic modification of a Tunisian variety of durum wheat known by its higher grain hardness, yield of gluten proteins, and minerals contained in endosperm [49].

During their growth, wheat plants are faced with various pathogens and unfavorable environmental conditions which negatively affect their yielding. Recently, the first *PR-1* gene was isolated from a durum wheat genome called *TdPR1.2*. This gene was activated upon plant treatment with exogenous SA application [31]. Moreover, TdPR1.2 physically interacted with the CaM/Ca^2+^ complex in a calcium-dependent manner. This interaction enhanced the catalytic activity of TdPR-1.2 in vitro. This activity was further enhanced in the presence of Mn^2+^ cations [31]. On the other hand, TdPR1.2 conferred abiotic stress tolerance (salt, osmotic, and heavy metal stress) to *E. coli* [31].

In this paper, we carried out a comprehensive genome-wide analysis of *PR-1* genes from durum wheat with bioinformatics analyses to boost our understanding of the molecular pathways of TdPR1 in stressful conditions and to enhance the exploitation of genetic resources in durum wheat. Structural analysis, the modeling of proteins, ligand–protein interactions, and expression profiling by qRT–PCR (under different stresses) were performed on the deduced TdPR1 proteins. These results highlight the crucial roles played by PR-1 proteins in *T. durum*, which provides clues towards their diversified roles in plants.

## 2. Results

### 2.1. Identification, Distribution, Gene Structures, and Conserved Motifs of PR-1 in Triticum durum

**Figure 1:**
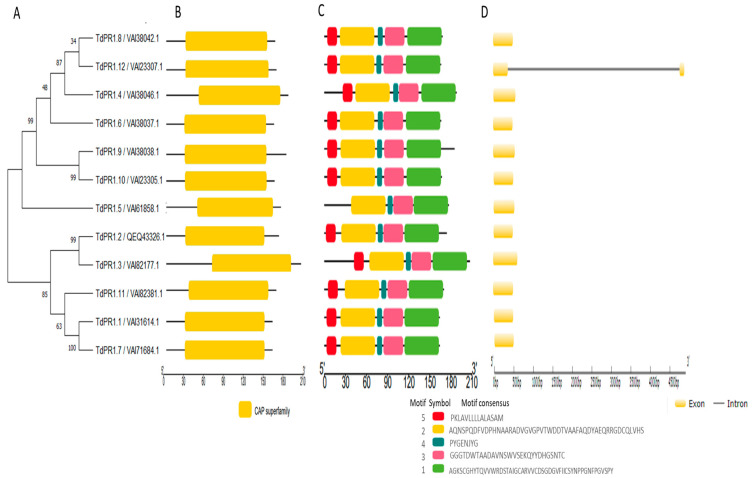
(**A**) Phylogenetic clustering performed by maximum-likelihood with MEGA 11, (**B**) CAP conserved domain represented by yellow color by using Tbtools v1.108, (**C**) Five conserved motifs represented by Tbtools v1.108, and (**D**) exon–intron structure of *PR-1* genes in *Triticum durum* genome by using gsds2.0.

After using BLASTp v 2.13.0 searches, the verification of the possession of CAP domain (cysteine-rich secretory proteins, antigen 5, and pathogenesis-related 1) (Figure 1), and the elimination of redundant sequences, twelve *TdPR1* sequences were obtained from the *Triticum durum* genome (named *TdPR1.1* to *TdPR1.12*). Moreover, the analyses of the exon–intron organization were performed to understand the evolution of the *TdPR1* genes. We found that the selected *PR-1* genes shared the same structure. In fact, only one gene (*TdPR1.12*) had two exons and one intron, whereas the other 11 *TdPR1* genes possessed a single exon. Furthermore, no 5′-UTR or 3′-UTR region was detected in the gene sequences (Figure 1). By using the Multiple Em for Motif Elicitation (MEME) (version 5.5.1), five motifs were identified. Four of them were presented in all the selected TdPR1 proteins (Motif 1, 2, 3, and 4), whereas motif 5 (presented by red boxes) was missing in TdPR1.5 (Figure 1).

**Figure 2:**
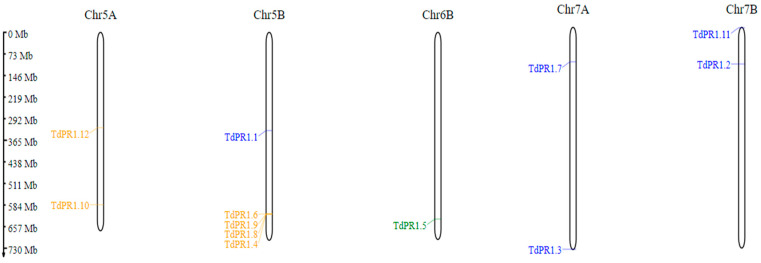
Chromosomal distribution of *PR-1* genes in *Triticum durum* genome, based on their groups I, II, and III. Gene ID are colored in orange, green, and blue, respectively, by using MG2C server v2.1.

The genomic features of *TdPR1* members are presented in Table 1. The distribution of *TdPR-1* genes were on five different chromosomes. Two genes were located on Chr5A, Chr7A, and Chr7B, whereas only one gene was located on chromosome 6B. The rest (about a third of the selected genes) were clustered on chromosome 5B (Figure 2, Table 1).

### 2.2. Multiple Alignment and Phylogenetic Relationship among the TdPR1 Genes

**Figure 3:**
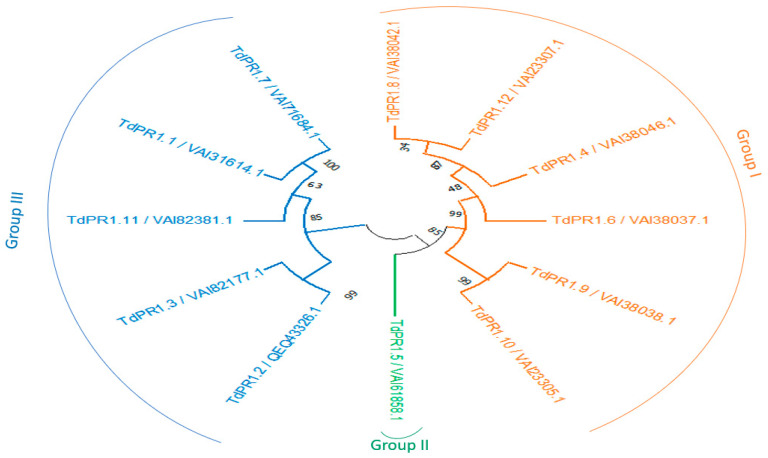
Phylogenetic reconstruction of PR-1 proteins from durum wheat. The phylogenetic tree was inferred to test maximum likelihood with 1000 bootstraps by MEGA 11. Group I present the acidic TdPR1 members in orange, Group II presents the basic TdPR1 members in green, and Group III presents the 2nd basic TdPR1 members in blue.

In order to understand the evolutional history of the candidate’s proteins, a phylogenetic tree was reconstructed. *TdPR-1* genes were distributed into three groups: Group I comprising genes encoding all acid proteins, Group III included five basic proteins and the six-basic protein, and the only TdPR1.5 located on Chr6B were clustered in Group II. Our findings show that this gene did not have the conserved motif 5 and had a different cleavage site sequence CNA-AF (Table 2; Figure 3).

**Figure 4:**
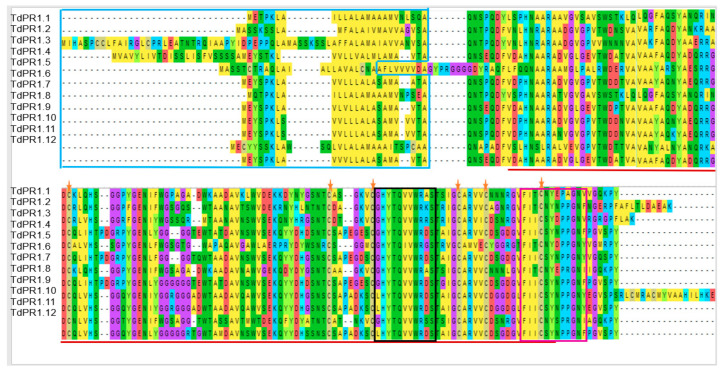
Multiple alignments of theTdPR-1 protein sequences. (blue rectangle: signal peptide; red line: CAP domain; orange star: conserved cysteine; black box: CRISP_1; and pink box: CRISP_2).

Based on the multiple alignment performed by the Muscle algorithm, we found that TdPR1 protein sequences are homologous. Indeed, the analysis indicated the presence of the Pfam0018 domain (CAP) in all the TdPR 1 proteins, (shown by red lines in the Figure 4). In addition, the blue box indicates the presence of the signal peptide (SP) regions (located at the N-terminal portion of the proteins; 24–31; Table 2). These peptides were found in all identified TdPR1 proteins except for TdPR1.3 and TdPR1.4. Moreover, only TdPR1.5 showed a cleavage site (between two alanine residues), whereas the others presented a common cleavage site between alanine (A) and glutamine (Q) amino-acid residues (Table 2).

According to the results given by the PROSITE database, *TdPR1* sequences contain two conserved domain CRISPs (cysteine rich secretory proteins) in the C-terminal: CRISP family signature 1 (CRISP_1) (shown by black box Figure 4;) and CRISP family signature 2 (CRISP_2) (shown as pink box Figure 4; Supplemental Figure S1).

### 2.3. Identification of Putative Zn^2+^ and CaM Binding Domains

**Figure 5:**
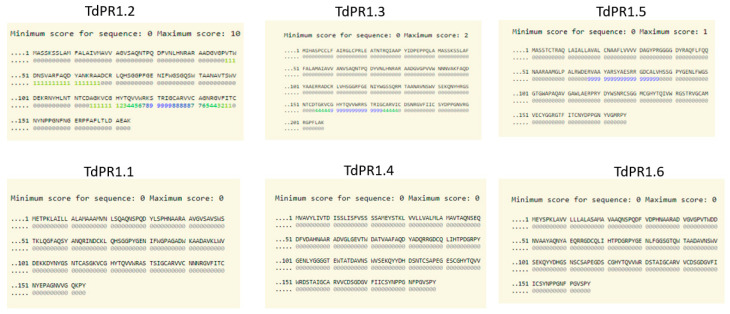
Identification of putative calmodulin binding domains in PR-1 proteins isolated from durum wheat using calmodulin target database.

We have recently identified a CaM binding domain located at the C-terminal part of the protein that ensures the interaction of the CaM/Ca^2+^ complex with the TdPR1 protein [31]. To investigate whether other identified TdPR1s harbor such a domain, we analyzed the structure of the 12 TdPR1 using the calmodulin target database. As revealed in Figure 5, only TdPR1.2, TdPR1.3, and TdPR1.5 harbor calmodulin binding domains at the C-terminal portion of the protein, whereas all the other TdPR1 proteins do not have such domains in their structures (Figure 5).

**Figure 6:**
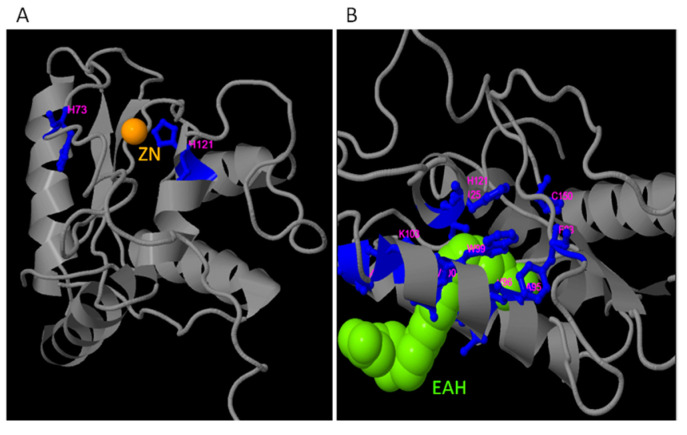
Localization of putative Zn (**A**) and EAH (**B**) binding sites in TdPR1.2 structure.

On the other hand, COACH analysis revealed the presence of three putative zinc ligand binding sites in the TdPR1.2 protein. In fact, ion (Zn^2+^) and a zinc molecule EAH ((5S, 7E, 9E, 11Z, 14Z)-5-hydroxyicosa-7, 9, 11,14-tetraenoic acid) were predicted. Different amino acids are implicated in this binding as revealed by the server which are 73 and 121 aa for the Zn ion (PDB Hit: 3mz8A) (Figure 6A) and 35,39,42,83,95,96,99,100,103,121,125,150 amino acids implicated in a possible interaction with the molecule EAH (PDB Hit: 3u3uC; Figure 6B).

### 2.4. Physicochemical Properties

Several analyses showed the different characteristics of the TdPR1 proteins listed in Table 3. In fact, the amino acid lengths of different proteins were between 164 and 207 aa with a molecular weight (MW) for all proteins ranging from 17 to 19 kDa. Among these proteins, 50% are acidic (pI < 7) and the others are basic (pI > 7). The negative GRAVY index (Grand average of hydropathicity) indicates that all TdPR1 proteins are non-polar (hydrophilic) proteins [50]. In addition, the thermostability parameter of proteins is measured by the aliphatic index (AI). The high aliphatic index of TdPR1 proteins, ranging from 64.46 to 76.76, indicates that they are thermo-stable under a wide temperature range [51].

### 2.5. Prediction of Transmembrane Helices on TdPR1

The topological analysis shows the existence of the transmembrane helixes in all the TdPR1 proteins, except TdPR1.7. In fact, the three online tools indicated the presence of transmembrane domains in TdPR1.2; TdPR1.5; TdPR1.9, and TdPR1.10. The results also showed that 66.66% of the online servers predicted that TdPR1.1, TdPR1.3, TdPR1.4, TdPR1.6, TdPR1.8, and TdPR1.12 have the transmembrane domain, whereas one out of three online tools indicated that this transmembrane helix is present in TdPR1.11 with a percentage of 33.33 (Table 4).

### 2.6. Predicted Secondary and 3D Structures of TdPR-1 Proteins

**Figure 7:**
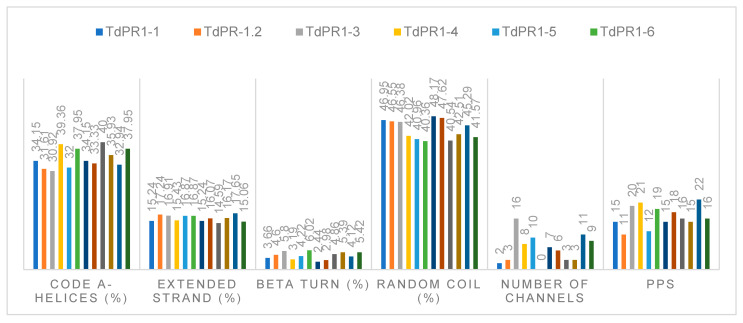
Percentage occurrence of the secondary structure analyses of TdPR-1 proteins. The different conformations of TdPR-1 proteins: α-helices, extended strands, β turns, and random coils were provided by SOPMA tool. BetaCavityWeb server was used to identify the putative numbers of channels. Predicted phosphorylation sites (PPS) were identified by NetPhos 3.1 server. PR-1 proteins were identified by different colors.

Secondary structures of proteins were predicted using the SOPMA server. The percentage of α-helices, extended strand, β turn, and random coil were 30.92–40, 14.59–18.08, 2.44–6.78, and 40.36–48.17, respectively. Structural conformation of a protein can occurs via protein phosphorylation by adding the phosphate groups to serine, threonine, or tyrosine residues [52]. Using the NetPhos 3.1 server, phosphorylation sites were predicted in TdPR1 proteins and ranged between 11 and 22 (Figure 7). The predicted channel number found in the studied TdPR1 proteins were registered between 0 and 16 (Figure 7).

**Figure 8:**
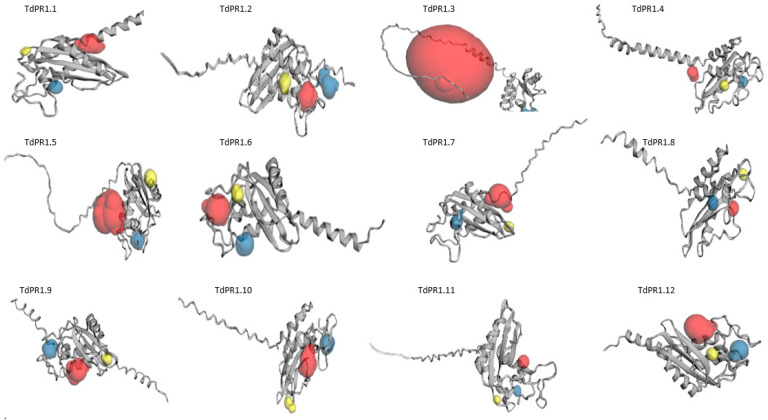
Predicted 3D structure of the TdPR1 proteins using the CASTp 3.0 server. The top three predicted pockets are indicated as red, blue, and yellow, respectively.

The predicted 3D structures of the TdPR1 proteins were constructed using the alpha fold online server. The 3D TdPR-1 proteins models presented four α-helices and several antiparallel β-sheets. According to the CASTp 3.0 analysis, molecular pockets were identified in all candidates. The top three predicted pockets, with the largest volume, are indicated as red, blue, and yellow, respectively (Figure 8).

### 2.7. In Silico Analysis of Cis-Elements

**Figure 9:**
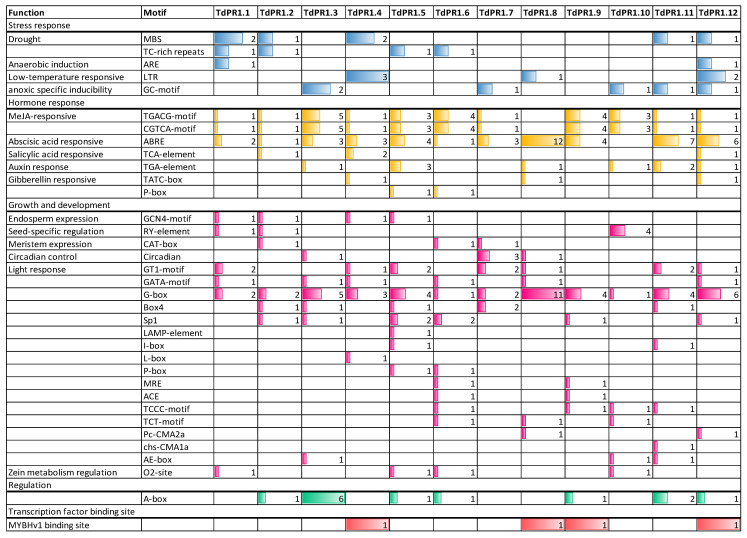
The number of cis-elements identified in the PR-1 promoter regions in Triticum durum. The cis-element numbers from each function response to stress, hormone, growth and development, regulation and transcription factors were represented by blue, yellow, pink, green and red, respectively.

**Figure 10:**
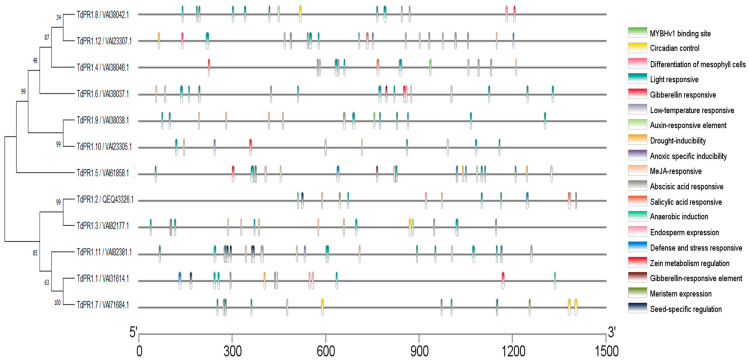
Representation of the cis-elements in the *TdPR1* promoters using TBtools software v1.108. Each cis-element was identified by a unique color.

The cis-acting element in the TdPR1 promoters could be divided into four categories, such as development-related elements, environmental stress-related elements, hormone-responsive elements, and transcription factor binding sites, based on the biological function analyzed by Plantcare (Figure 9 and Figure 10). Stress responsive cis-elements were found in most of the *PR-1* promoters except for *TdPR1.9*. Cis-regulatory elements belonging to this class are associated with drought, anaerobic anoxic specific inducible element, and low-temperature response. All *TdPR1* regulatory regions have more than four cis-element hormone responses. The MeJA- and abscisic acid responsive motifs are the most abundant in most of *TdPR1*. Thus, ABRE (abscisic acid response elements) are the elements which had the highest motif number in the *TdPR1* promoter region (12 motifs in *TdPR1.8*). Salicylic acid, auxin, and gibberellin responsive elements were found in some *TdPR1* promoters. Moreover, different cis-acting regulatory elements involved in growth and development are present. The most abundant motifs are related to light response as all the identified TdPR1 proteins presented a diversity of light response elements. G-box is the only motif present in all the identified promoters with a maximum number registered for *TdPR1.8* (11 G-boxes). In addition, endosperm expression, seed-specific regulation, meristem expression, and circadian control are found in the majority of PR-1 in *Triticum durum*. The binding domains of a single TF family, MYB, were identified in four, eight, nine, and twelve *TdPR1* promoters.

### 2.8. Predicting TdPR1 Pproteins Subcellular Localization

**Figure 11:**
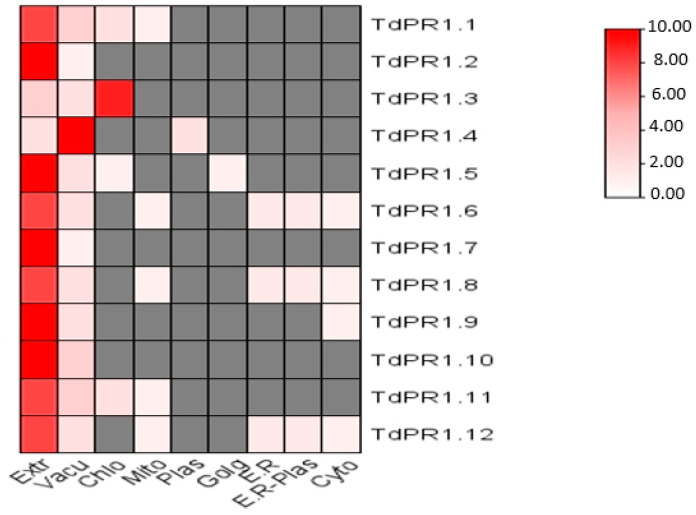
Heatmap of the subcellular localization prediction of the TdPR1 through WoLF PSORT using Tbtools v1.108. This graphic represents the prediction of subcellular localization in the different compartments (Extr: extracellular, Vacu: vacuole, Chlo: chloroplast E.R: endoplasmic reticulum, E.R. plas: endoplasmic reticulum plasma membrane, Cyto: cytoplasm, Mito: mitochondria). The intensity of color correlates with the subcellular localization prediction.

The subcellular localization of TdPR1 proteins performed by the online tool WoLF PSORT and represented by a heatmap graphic using Tbtools v1.108 are shown in Figure 11. Indeed, with a high level of prediction, 10 TdPR1 proteins were found to be located in the extracellular compartment. This subcellular location of PR1 is not only identified in the extracellular space but could also be in the extracellular, Vacuole, endoplasmic reticulum, endoplasmic reticulum plasma membrane, cytoplasm, and mitochondria.

### 2.9. Gene Ontology (GO) Term Distribution of Triticum durum PR-1

**Figure 12:**
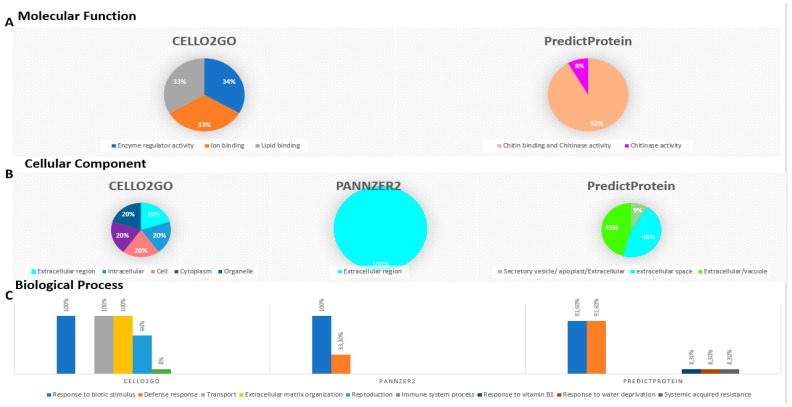
Comparative analysis of gene ontology (GO) by using three different online tools: CELLO2GO, PredictProtein, and PANNZER2. (**A**) Prediction of molecular function of TdPR1, (**B**) Cellular Component of PR1, and (**C**) Biological process predicted for the identified proteins.

Gene ontology (GO) analyses performed by three different servers showed variable results on biological process, molecular function, and cellular component terms for 12 TdPR1 proteins (Figure 12). Based on CELLO2GO data, three molecular functions were identified: enzyme regulator activity, ion binding, and lipid binding. The PredictProtein tool predicted that 8% of the deduced PR1 had only a chitinase activity and 11 out of 12 (91.6%) had both functions: chitinase activity and chitin binding (Figure 12A). No result was obtained with PANNZER2. In terms of cellular components, CELLO2GO, PANNZER2, and PredictProtein identified that TdPR1 proteins could be located in the extracellular region with a percentage of 20, 100, and 46, respectively (Figure 12B). The biological processes regulated by all TdPR1 proteins (91.6–100%) were the response to biotic stimulus (GO:0009607) identified by the different web servers. Based on the GO enrichment analysis by PANNZER2 and PredictProtein servers, most of the *PR-1* genes are implicated in defense responses (GO:0006952) (33.3%, 91.6%, respectively). Four other biological processes were found in CELLO2GO, namely transport (100%), extracellular matrix organization 100%), reproduction (66.6%), and immune system process (8.3%). Moreover, three other Gene Ontology terms were identified by the PredictProtein tool in one out of twelve TdPR1 proteins (8.3%): systemic acquired resistance (GO:0009627), response to water deprivation (GO:0009414), and response to vitamin B1 (GO:0010266) (Figure 12C).

### 2.10. Differential Expression of TdPR1.2 Gene under Various Stress Conditions

**Figure 13:**
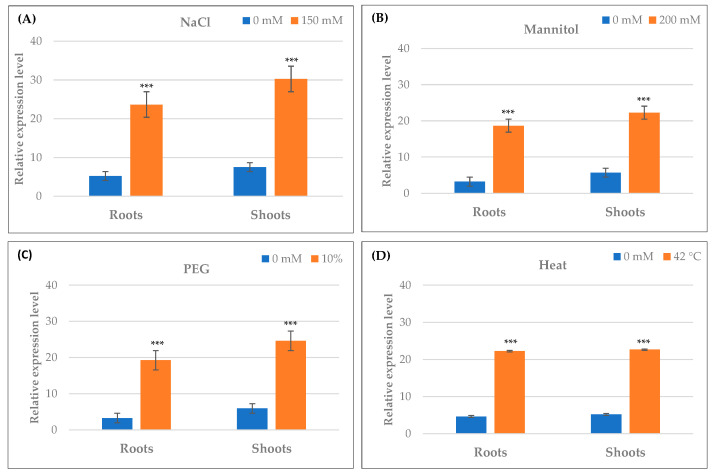
qRT–PCR expression analysis of *TdPR1.2* gene under different abiotic stresses, (**A**) salt, (**B**) mannitol, (**C**) PEG 10%, and (**D**) heat. (***) indicates value significantly different from the control. Statistical significance was assessed by applying the Student *t*-test at *p* < 0.01.

**Figure 14:**
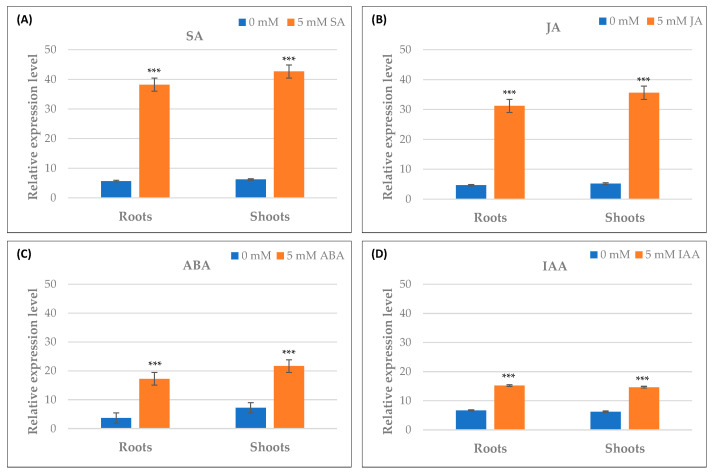
qRT–PCR expression analysis of *TdPR1.2* gene under different hormonal stresses, (**A**) salicylic acid (SA), (**B**) jasmonic acid (JA), (**C**) abscisic acid (ABA), and (**D**) IAA. (***) indicates value significantly different from the control. Statistical significance was assessed by applying the Student *t*-test at *p* < 0.01.

The full-length cDNA sequence of *TdPR1.2* (GenBank accession no. MK570869.1) was previously identified by our group [31]. To investigate the possible biological functions of the *TdPR1.2* gene, we assessed the expression patterns of *TdPR1.2* genes in wheat under various abiotic stress conditions using qRT–PCR (Figure 13 and Figure 14).

In response to salt stress (150 mM NaCl), TdPR1.2 was significantly upregulated (Figure 13A). The same result was observed when plants were subjected to mannitol and PEG stresses (Figure 13B,C). When heat stress was applied to wheat plants (42 °C for 30 min), there was a significant increase in TdPR1.2 expression level in the roots and shoots, suggesting that this protein could have a putative protective role in controlling wheat heat tolerance (Figure 13D).

The hormonal response of *TdPR1.2* gene was investigated by treating plants with salicylic acid (SA), indole acetic acid (IAA), jasmonic acid (JA), and abscisic acid (ABA). As shown in Figure 14, TdPR1.2 was upregulated in response to all hormones used in this work. Overall, these results demonstrate that TdPR1.2 is implicated in plant response to many abiotic and hormonal stresses.

## 3. Discussion

Different studies have shown that PR1 proteins play a crucial role in plants’ responses to different diseases that affect common wheat. In fact, the overexpression of TaPR1-7 enhanced plant resistance to infection by *Puccinia striiformis* f. sp. tritici (Pst) (Stripe (yellow) rust) [53], while TcLr19PR1 [54] and TaLr35PR1 genes [55] were induced after plant infection with *Puccinia triticina* (leaf rust) attack. Meanwhile, TaPR1a was highly expressed and caused plant resistance to both diseases (stripe (yellow) rust and leaf rust) [56]. Previously, we have demonstrated that the newly isolated gene, TdPR1.2, presented an antibacterial and antifungal activity in vitro. Interestingly, TdPR1.2 presented a positive effect in inhibiting *Septoria tritici* growth in vitro [31]. Moreover, pathogenesis-related protein 1 could positively interact with other PR families. Wang et al. [57] found that TaTLP1 and TaPR1 interacted physically to protect wheat plant from leaf rust.

Furthermore, PR protein families were activated by *Septoria tritici* blotch disease. For instance, PR1 and PR3 were upregulated in Sevin cultivar after plant infection with *Septoria tritici* [58], whereas in Wangshubai, it has been demonstrated that such infection causes the upregulation of PR1 and peroxidase genes [59]. Finally, *Septoria tritici* infection induced the upregulation of PR-1 in Seri 82 and Frontana cultivars, respectively [60].

While different studies have investigated the role of TaPR1 proteins in plants, little is known about its homologue in durum wheat, TdPR1.2. Therefore, the genetic richness of durum wheat *PR-1* genes, its molecular functions in response to abiotic and biotic stresses, is a crucial topic to understand and resolve, not only for the agronomic problems of wheat, but also for the economics and marketing of the country.

Several researchers are emphasising the identification and characterization of PR-1 proteins in different plants. They state that most PR-1s are often encoded by multi-gene families as identified in many plant species such as black pepper [19], tomato [7], and the two genomes of the banana plant [24]. In this work, we identified 12 PR-encoding genes in durum wheat genome which is less than the number of genes identified in common wheat [18], soybean [20], rice, and *Arabidopsis thaliana* [16] suggesting that there is no correlation between the plant genome size and the identified PR-1 members [24]. As revealed in this work, the twelve identified *TdPR1* genes were distributed on five chromosomes. Two genes were present on Chr5A, Chr7A, and Chr7B, whereas only one gene was located on chromosome 6B. The rest (about a third of the selected genes) were clustered in chromosome 5B (Figure 2, Table 1). Liu and Xue (2006) [61] explained the clustering of genes in one chromosome by the tandem gene duplication events which could occur during evolution.

A phylogenetic tree was performed by the MEGA11 software. The *TdPR1* genes were subdivided into three phylogenetic groups: Group 1 (six acidic TdPR1), Group 2 (one basic TdPR1) and Group 3 (five basic TdPR1). The division of pathogenesis-related protein-1 genes into three groups based on protein isoforms (acidic/basic) have also been shown in previous studies, such as in rice [61]. The same result was found in common wheat, which has one of the biggest genomes (>16,000 megabases) among monocot agricultural plants and presented 23 *PR-1* genes. The 23 *TaPr-1* genes all have intron-free open reading frames that express a signal peptide at the N-terminus and a conserved PR-1-like domain, according to a sequence study of the genes. According to phylogenetic analysis, *TaPr-1* genes, along with their counterparts in other monocots, form three major monophyletic groups; each group contains genes that encode basic, basic with a C-terminal extension, and acidic PR-1 proteins, respectively. This suggests that the functions of *PR-1* genes in monocot plants are diverse and conserved [18]. In contrast, in sugarcane and tomato plants, pathogenesis-related proteins were clustered in the same group independently of their type (basic/acid) [7,23]. The gene structure analyses identified the presence of a single gene and a lack of intron in all *TdPR1* except *TdPR1.12* which presented two exons. In accordance with other plants, such as sugarcane (*Saccharum spontaneum)* [23], tea plant (*Camellia sinensis* (L.) *O. Kuntze*) [17], and banana (*Musa balbisiana* (*DH-PKW*)) [24], the majority of genes had only one exon and lacked introns (13 out of 19 *ScPR1*, 15 out of 17 *CsPR1*, and 10 out of 11 *MaPR1*, respectively). Previously, scientists have claimed that, during evolution, genes which were activated rapidly to respond to stresses were likely to decrease their intron density [62]. Zhang and his colleagues found that the expression of the *CsPR1* gene, which does not have introns, were expressed more rapidly than genes with three introns in plants subjected to blight disease stress [17].

The majority of motifs are conserved among all TdPR1 proteins. The sequence alignment of the deduced protein presents two conserved domains belonging to the CRISP family. Previous studies have demonstrated that CRISPs may be involved in the plant to acquire resistance to biotic and abiotic stresses [63]. Moreover, the first isolated TdPR1.2 was aligned with eight PR-1 sequences from different species, demonstrating that the cysteine residues and the CRISP_1 domain are highly conserved in both monocotyledonous and dicotyledonous plants, whereas CRISP_2 is less conserved [11,31]. Moreover, all PR-1 proteins presented six well conserved cysteines residues [11,31,61]. These results are consistent with our findings and all the identified *TdPR1* genes presented the conserved residues (Figure 1), suggesting a common molecular function of all identified PR-1 genes. In addition, the analysis of the *TdPR1* gene structure and the conserved motifs indicates that TdPR1 genes are highly conserved, as demonstrated by the number of α and β helices and by the presence of signaling peptides. Such findings suggest that all the encoding proteins present the same function under environmental stress [19,24,64].

Numerous cis-elements were identified in *TdPR1* genes (Figure 10) involved in stress, development, and hormone response. Additionally, the MYB transcription factors (TFs), who had a detectable binding domain in four *TdPR1* genes, have a different role, such as plant growth and development, physiological activity, primary and secondary metabolic reactions, and responses to biotic and abiotic stresses [65], suggesting the implication of these proteins in plant metabolism and development. Since our promoter regions possess a myriad of cis-elements, we suggest that the identified genes could be implicated in different molecular and metabolic pathways.

The signal peptide plays an important role in the guiding of proteins into subcellular spaces [66]. Based on the results obtained by the WoLF PSORT web server, the two proteins which do not contain the signal peptide (TdPR1.3 and TdPR1.4) are more probably localized in chloroplast and vacuole, respectively. The *PR-1* gene family identified in other plants may prove our suggestion. In fact, scientists have stated that pathogenesis-related proteins in grape and wheat containing the signal peptide could be secreted into the extracellular compartment [17,18,21]. However, in tea (*Camellia sinensis* (L.) *O. Kuntze*) and black pepper (*Piper nigrum*), PR-1 proteins without SPs were found in the intracellular compartment [17,19]. The acquisition of SP at the N terminal sequences might be affecting the subcellular localization of proteins. Therefore, TdPR1 proteins containing the signal peptide at the N terminal assume their guidance into the extracellular compartment in order to guarantee their cellular functions.

Putative ligand prediction shows that TdPR1.2 possess binding sites to Zn^2+^ and EAH, similarly to the HbPR-1 protein from the rubber tree (*Hevea brasiliensis*), which had imbibed in its structure two binding sites of those ligands plus a glycerol binding domain [67]. Interestingly, no glycerol binding sites were detected in TdPR1.2 proteins.

Recently, a CaM binding domain was identified by our group and in the C-terminal part of the TdPR1.2 [31]. TdPR1.2 interacts with CaMs in a calcium-dependent manner and this interaction enhances TdPR1.2 activity, especially in the presence of Mn^2+^ cations [31]. To further identify whether the other identified *TdPR1* genes harbors a CaMBD in their structures, we performed in silico analysis using calmodulin target databases. In the sequence of three different PR proteins (PR1.2; PR1.3; and PR1.50), putative calmodulin-binding domains were identified. In these proteins, this domain is located at the C-terminal portion of the protein sequence. It was noted that this domain is necessary for calmodulin binding and the calcium-dependent activation of various plant proteins such as catalases [68,69], PRs [31], and mitogen-activated protein kinase phosphatases [70,71].

Calmodulins (CaMs) are omnipresent, tiny proteins with only four Ef-Hand motifs, which are common components of Ca^2+^ binding proteins [68]. The main intracellular Ca^2+^ signaling pathways are mediated by CaMs, and an increase in the amount of Ca^2+^ in the nucleus or cytosol leads to the formation of Ca^2+^/CaM complexes, which interact with a wide range of targets, including ion transporters, protein kinases, pathogen-related proteins, transcription factors, and protein phosphatases, and control cellular functions [9,70,72,73].

In this study, TdPR1.2 was upregulated in the leaf and root tissues of durum wheat subjected to NaCl (150 mM) and PEG (10% PEG 6000) treatments as previously shown in other studies such as in *Zea mays* (*ZmPR-1*; [74]), banana [24], tomato (13 *SlPR-1* genes; [7]), *Vitis vinifera* (*VvPR-1*; [75]), and rice (*OsPR1a*; [76]), suggesting that this genes could have a dual role depending on the tissue expression in the plant.

It has been described in the literature that the SA and JA signaling pathways are stimulated after biotrophic/hemibiotrophic (under the control of SA) and necrotrophic (under the control of JA) pathogen infection [5]. Thus, we investigated the effect of SA and JA application on *TdPR1.2* gene expression in wheat. Our results showed that TdPR1.2 was upregulated after application of those phytohormones in the roots and shoots of wheat. Other PR-1 proteins were reported to be upregulated after plant treatment with SA and JA [7,77]. In banana, MaPR1-1 was upregulated after plant treatment with SA and JA stresses due to the presence of cis-elements and binding sites for transcription factors [24]. Thus, the identification of the stress-responsive elements involved in the up/downregulation of PR-1 will help in understanding plants’ resistance mechanisms toward various stresses. These findings strongly suggest that the *TdPR1.2* gene plays a crucial role in plant defense against environmental stresses. It has been suggested that *PR-1* genes can serve as molecular markers associated with resistance to different biotic and abiotic stresses [5,19,78]. Thus, our findings could be useful for breeding programs aimed at increasing the resistance of wheat crops to salt, drought, and hormonal stresses as well as plant infection with pathogens.

## 4. Materials and Methods

### 4.1. Identification of PR-1 Genes from the T. durum Genome

In order to identify the genomic family members of the *PR-1* genes, the Pfam’s CAP domain (PF00188), downloaded from the Pfam database version 35.0 (https://pfam.xfam.org/, accessed on 5 November 2022) [79], was used as a query for search against the *Triticum durum* genome (taxid:4567) by using Blastp program v 2.13.0. After the realisation of the homology search, only predicted PR-1 sequences with an e-value <10–10 were selected. The redundancy in the resulted sequence’s collection was removed by the decrease in redundancy (https://web.expasy.org/decrease_redundancy/; accessed on 8 November 2022) program. Selected sequences were then analysed using SMART database (http://smart.embl-heidelberg.de/, accessed on 8 November 2022) [80], PfamScan (https://www.ebi.ac.uk/Tools/pfa/pfamscan/; accessed on 8 November 2022) [81] and NCBI’s Conserved Domain Search v3.20 (http://www.ncbi.nlm.nih.gov/cdd/; accessed on 8 November 2022) [82]. The selected TdPR1 sequence possession of the conserved CAP domain was drawn by Tbtools v1.108 (Graphi [83]).

### 4.2. Sequence Analysis

Physiochemical properties of TdPR1 proteins were calculated with ProtParam program (https://web.expasy.org/protparam/; accessed on 12 November 2022) [84]. The possession of the signal peptide for the protein sequences was identified by SignalP 5.0 Server (https://services.healthtech.dtu.dk/service.php?SignalP-5.0; accessed on 13 November 2022) [85]. The presence of Zinc binding domain was revealed by COACH web server to determine the putative ligands abled to interact in the binding site of TdPR1.2 protein [86,87]. The presence of putative calmodulin binding sites was also revealed by Calmodulin target database (http://calcium.uhnres.utoronto.ca/ctdb/pub_pages/general/index.htm; accessed on 21 December 2022) [88].

### 4.3. Topological Analysis

The numbers of the transmembrane helices of each PR proteins were predicted by using various online servers such as TMHMM-2.0 (https://services.healthtech.dtu.dk/service.php?TMHMM-2.0; accessed on 5 January 2023) [89], SOSUI version 1.11 (http://harrier.nagahama-i-bio.ac.jp/sosui; accessed on 2 January 2023) [90], and TMDET version 2.0 (http://tmdet.enzim.hu/; accessed on 3 January 2023) [91].

### 4.4. Secondary and Tertiary Structure Prediction

By using SOPMA online server (Self-Optimized Prediction Method with Alignment) (https://npsa-prabi.ibcp.fr/cgi-bin/npsa_automat.pl?page=/NPSA/npsa_sopma.html; accessed on 26 December 2022), the secondary structure of the TdPR-1 proteins was predicted [92]. The putative number of the predicted phosphorylation sites in TdPR1 were identified by NetPhos 3.1 server (http://www.cbs.dtu.dk/services/NetPhos/; accessed on 5 January 2023) [93,94]. The 3D structures of the TdPR-1 proteins were predicted using the Alphafold online server v2. 0 (https://alphafold.ebi.ac.uk/; accessed on 27 December 2022) [95,96]. The BetaCavityWeb server (http://voronoi.hanyang.ac.kr/betacavityweb; accessed on 10 January 2023) was used to predict the number of channel structural [97]. The CASTp 3.0 (Computed Atlas of Surface Topography of proteins) online server (http://sts.bioe.uic.edu/castp/calculation.html; accessed on 27 December 2022) was used to predict the active site pockets of the TdPR-1 protein [98].

### 4.5. Conserved Motif, Multiple Alignment, and Phylogenetic Tree

ScanProsite online server were used to scan Protein domains and functional sites (http://prosite.expasy.org/prosite.html; accessed on 10 November 2022) [99] and the logo of the motifs signature was drawn by WebLogo version 2.8.2 (https://weblogo.berkeley.edu/logo.cgi; accessed on 14 January 2023) [100,101]. MEME server v5.1.1 (http://meme-suite.org/tools/meme; accessed on 12 November 2022) online tool were used to predict the conserved motif in TdPR1 [102], then the motifs were visualized by TBtools software v1.108 (http://github.com/CJ-Chen/TBtools) [83]. TdPR1 protein sequences were aligned by a multiple sequence alignment using MUSCLE program (accessed on 12 November 2022) [103], and the phylogenetic tree of the aligned TdPR-1s sequences was constructed with the Maximum-Likelihood and 1000 bootstrap values using the MEGA11 software (accessed on 12 November 2022) [104].

### 4.6. Cis-Elements, Chromosomal Locations, and Gene Structure Analyses

Promoters were identified as the 1.5 kb upstream the start codon region, and were downloaded from NCBI database (http://www.ncbi.nlm.nih.gov/; accessed on 23 January 2023). Analysis of cis-acting regulatory elements of promoter sequences were performed by PlantCARE online tools (http://bioinformatics.psb.ugent.be/webtools/plantcare/html/; accessed on 23 January 2023) [105] and visualized by TBtool software v1.108 (accessed on 23 January 2023) [83]. The chromosome length data were collected from the NCBI and used to visualize the genomic distribution of PR1 genes in durum wheat by using the online software MG2C v2.1 (http://mg2c.iask.in/mg2c_v2.1/; accessed on 19 November 2022) [106,107]. The identified *PR-1* genes and coding sequences (CDS) features were obtained from NCBI, then their exon-intron structures were retrieved using the Gene Structure Display Server program 2.0 (http://gsds.gao-lab.org/; accessed on 19 November 2022) [108].

### 4.7. Subcellular Localization and Gene Ontology Analysis

Subcellular location was predicted using WoLFPSORT online tool (http://wolfpsort.seq.cbrc.jp/; accessed on 27 January 2023) [109]. Gene ontology (GO) analysis was performed by three different online tools, such as PANNZER2 (http://ekhidna2.biocenter.helsinki.fi/sanspanz/; accessed on 27 January 2023) [110], CELLO2GO (http://cello.life.nctu.edu.tw/cello2go/; accessed on 27 January 2023) [111], and PredictProtein (http://ppopen.rostlab.org; accessed on 27 January 2023) [112].

### 4.8. Plant Material and Stress Treatments

In this work, seeds of wheat (*Triticum durum* Desf.) (cv. Om Rabiaa) were sterilized in each box containing 30 mL of 0.6% NaClO solution for 15 min, then washed five times with 50 mL sterile water. For each treatment, 45 seeds were placed in each Petri dish (11 cm long, 2.5 cm high, and 11 cm wide) in the presence of a sponge and filter paper placed below to maintain moisture at 25 ± 2 °C. Seeds were then transferred to a greenhouse at 24 ± 2 °C, with photosynthetically active radiation of 280 μmol m^−2^ s^−1^, a 16 h photoperiod, and 60 ± 10% relative humidity. After 10 days, seedlings were subjected to stresses. In this study, nine treatments were used including the control (distilled water), 150 mM NaCl, 10% PEG, 200 mM mannitol, 5 mM of each phytohormone (SA, JA, IAA, and ABA), and heat (42 °C). Each treatment was replicated three times. Finally, shoots were harvested and immediately frozen in liquid nitrogen and stored at −80 °C.

### 4.9. RNA Extraction and Quantitative Real-Time Reverse Transcription PCR (qRT-PCR)

Total RNA was extracted from individual roots and leaves (0.5 g of each tissue) using the RNeasy Plant Mini Kit (QIAGEN, Hilden, Germany). Extracted RNA was then purified from genomic DNA (RNase free DNase set; QIAGEN), qualified by gel electrophoresis, and used for first-strand cDNA synthesis (GoScript Reverse Transcription System; Promega, Madison, USA) with an oligo-dT primer. PCR reactions were achieved in a 10 μL final volume tube in the presence of 3 μL cDNA (obtained from 40 ng of DNase-treated RNA), 0.5 μL of each primer of the *TdPR1.2* gene at 10 μM (TdPR_Fw: 5′-ATGGCATCT TCCAAGAGT-3′ and TdPR_Rv: 5′-TCA AGG GTG AGG ACG CGA A-3), 5 μL 2 × SYBR Green I master mix, and 1 μL of RNase-free water (Sigma). The reaction consisted of an initial denaturation at 95 °C for 5 min followed by 40 cycles composed of 10 s at 95 °C, 20 s at 60 °C, and 30 s at 72 °C, then a melting curve (5 s at 95 °C, 1 min at 65 °C, and 5 min with the temperature increasing from 65 to 97 °C). Three biological repetitions were performed for each experimental condition, with three technical repetitions for each sample. Melting curve analysis at the end of cycling was used to verify whether there was single amplification. At the end of the reaction, the threshold cycle (CT) values of the triplicate PCRs were averaged and used for transcript quantification. The relative expression ratio of the *TdPR1.2* gene was calculated by using the comparative CT method with the *actin* gene designed from the *T. aestivum* genome (actin_Fw: 5′-TCC CTC AGC ACA TTC CAG CAGAT-3 and actin_Rv: 5′-AAC GAT TCC TGG ACC TGC CTC ATC-3′) as an internal expression standard [113]. The relative expression level was calculated from triplicate measurements based on the 2^−ΔΔCT^, where ΔΔCT = (CT, target gene−CT, actin) stressed − (CT, target gene−CT, actin) control. Relative expression ratios from three independent experiments (three biological repetitions) are reported.

### 4.10. Statistical Analysis

Data are reported as mean ± S.E. The results were compared statistically by using Student’s *t* test, and differences were considered significant at *p* < 0.01.

## 5. Conclusions

PR 1 proteins play important roles in growth regulation, development, and plant response to biotic and abiotic stress. Here, plenty of in silico tools were used to enhance our comprehensive understanding of the PR-1 family in *Triticum durum* plants. In fact, twelve TdPR1 were identified and clustered into three phylogenetic groups. Gene structure, conserved motifs, and physicochemical properties showed that TdPR1 proteins present highly conserved structures. Moreover, a myriad of cis-elements in the up/down stream of *TdPR1* genes were found and may act in the gene expression to stress responses, hormones, and growth in durum wheat. Following cis-regulatory elements of the promoter regions, TdPR1 are clustered on chromosomes Chr5A, Chr5B, Chr6B, Chr7A, and Chr7B. All the predicted PR1 proteins identified in this work were reported to be extracellular proteins. Collectively, the findings of this study will contribute to a better understanding of molecular mechanisms and provide useful and essential information for the further functional characterization of TdPR-1 genes in future research.

## Figures and Tables

**Table 1:** Features of PR-1 genes identified in *Triticum durum*.

Gene Name	Locus/Protein Id	Gene Identifier/ ORF Names/Locus Tag	Chr	Strand	EMBL ID	Start	End	N° Exon
TdPR1.1	VAI31614.1	TRITD5Bv1G112400	Chr5B	−	LT934120.1	330,646,842	330,647,336	1
TdPR1.2	QEQ43326.1	TRITD_7Bv1G043610	Chr7B	+	MK570869.1	120,612,592	120,613,116	1
TdPR1.3	VAI82177.1	TRITD_7Av1G281080	ChrA	−	LT934123.1	727,376,831	727,377,454	1
TdPR1.4	VAI38046.1	TRITD_5Bv1G217120	Chr5B	+	LT934120.1	613,827,887	613,828,453	1
TdPR1.5	VAI61858.1	TRITD6Bv1G202080	Chr6B	+	LT934122.1	630,324,650	630,325,183	1
TdPR1.6	VAI38037.1	TRITD_5Bv1G216890	Chr5B	+	LT934120.1	613,290,494	613,290,994	1
TdPR1.7	VAI71684.1	TRITD7Av1G051340	Chr7A	+	LT934123.1	113,022,659	113,023,153	1
TdPR1.8	VAI38042.1	TRITD5Bv1G217000	Chr5B	−	LT934120.1	613,580,189	613,580,695	1
TdPR1.9	VAI38038.1	TRITD_5Bv1G216900	Chr5B	+	LT934120.1	613,295,645	613,296,202	1
TdPR1.10	VAI23305.1	TRITD_5Av1G219320	Chr5A	+	LT934119.1	582,431,569	582,432,072	1
TdPR1.11	VAI82381.1	TRITD_7Bv1G001260	Chr7B	−	LT934124.1	2,277,012	2,277,524	1
TdPR1.12	VAI23307.1	TRITD_5Av1G219360	Chr5A	+	LT934119.1	582,698,401 582,703,182	582,698,778 582,703,304	2

**Table 2:** Signal peptide region detected from the TdPR-1 proteins.

Gene Name	Locus/Protein Id	Cleavage Site Position	Sequence Position	Probability	Signal Peptide (Sec/SPI)	Other
TdPR1.1	VAI31614.1	24–25	SQA-QN	0.8626	0.996896	0.003104
TdPR1.2	QEQ43326.1	25–26	VSA-QN	0.9374	0.997416	0.002584
TdPR1.3	VAI82177.1	-	-	-	0.089198	0.910802
TdPR1.4	VAI38046.1	-	-	-	0.260482	0.739518
TdPR1.5	VAI61858.1	23–24	CNA-AF	0.5384	0.791296	0.208704
TdPR1.6	VAI38037.1	23–24	ATA-QN	0.4766	0.9964	0.0036
TdPR1.7	VAI71684.1	24–25	SEA-QN	0.8419	0.998760	0.001240
TdPR1.8	VAI38042.1	23–24	VTA-QN	0.5392	0.993040	0.006960
TdPR1.9	VAI38038.1	24–25	VTA-QN	0.8174	0.994069	0.005931
TdPR1.10	VAI23305.1	24–25	VTA-QN	0.6873	0.995200	0.004800
TdPR1.11	VAI82381.1	30–31	CAA-QN	0.5826	0.975437	0.024563
TdPR1.12	VAI23307.1	23–24	VTA-QN	0.5527	0.991300	0.008700

**Table 3:** Physio-chemical characteristics of TdPR1 using Protparam ProtParam software v 3.1 (https://web.expasy.org/protparam/).

Gene Name	Locus/Protein Id	Length	MW	pI	Aliphatic Index	Gravy
TdPR1.1	VAI31614.1	164	17,634.89	8.74	72.07	−0.236
TdPR-1.2	QEQ43326.1	174	18,836.12	9.02	65.11	−0.238
TdPR1.3	VAI82177.1	207	22,622.68	9.41	72.66	−0.229
TdPR1.4	VAI38046.1	188	20,172.30	4.23	76.76	−0.043
TdPR1.5	VAI61858.1	177	19,162.68	8.83	65.20	−0.106
TdPR1.6	VAI38037.1	166	17,702.37	4.47	64.10	−0.330
TdPR1.7	VAI71684.1	164	17,592.72	8.21	70.30	−0.230
TdPR1.8	VAI38042.1	168	17,921.57	4.28	64.46	−0.320
TdPR1.9	VAI38038.1	185	19,868.14	5.18	73.84	−0.186
TdPR1.10	VAI23305.1	167	17,801.64	4.82	70.12	−0.265
TdPR1.11	VAI82381.1	170	18,449.72	7.53	71.24	−0.062
TdPR1.12	VAI23307.1	166	17,657.44	4.58	68.19	−0.184

**Table 4:** Topological analysis of PR proteins using different online tools. (0—absent; 1—Present).

Gene Name	Locus/Protein Id	TMHMM	SOSUI	TMDET	Percentage (%)
TdPR1.1	VAI31614.1	0	1	1	66.66
TdPR-1.2	QEQ43326.1	1	1	1	100
TdPR1.3	VAI82177.1	0	1	1	66.66
TdPR1.4	VAI38046.1	0	1	1	66.66
TdPR1.5	VAI61858.1	1	1	1	100
TdPR1.6	VAI38037.1	0	1	1	66.66
TdPR1.7	VAI71684.1	0	0	0	0
TdPR1.8	VAI38042.1	0	1	1	66.66
TdPR1.9	VAI38038.1	1	1	1	100
TdPR1.10	VAI23305.1	1	1	1	100
TdPR1.11	VAI82381.1	0	1	0	33.33
TdPR1.12	VAI23307.1	0	1	1	66.66

## Data Availability

The data generated and analyzed during this study are included in this article.

## Supplementary Materials

The following supporting information can be downloaded at: https://www.mdpi.com/article/10.3390/plants12101998/s1, Figure S1: The two CRISP domains conserved region signature generated by WebLogo version 2.8.2. (A) CRISP-1 logo and (B) CRISP-2 logo signature.
