# Supplementary figures and images for: Genome-Wide Identification and Expression Profiling of Pathogenesis-Related Protein 1 (PR-1) Genes in Durum Wheat (Triticum durum Desf.)
Source: Plants (Basel). 2023 May 16;12(10):1998. doi: 10.3390/plants12101998 (PMC10223549; doi:10.3390/plants12101998)

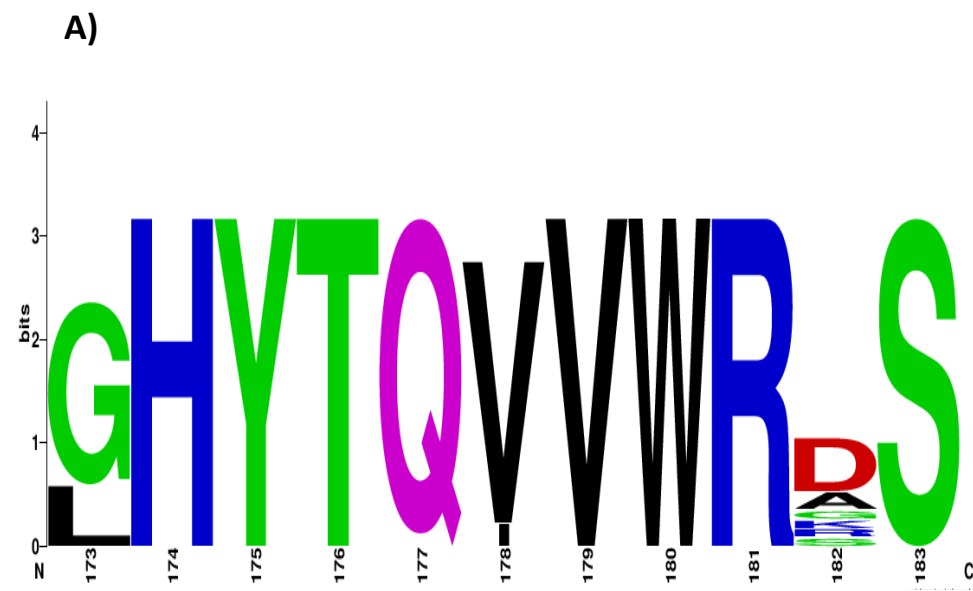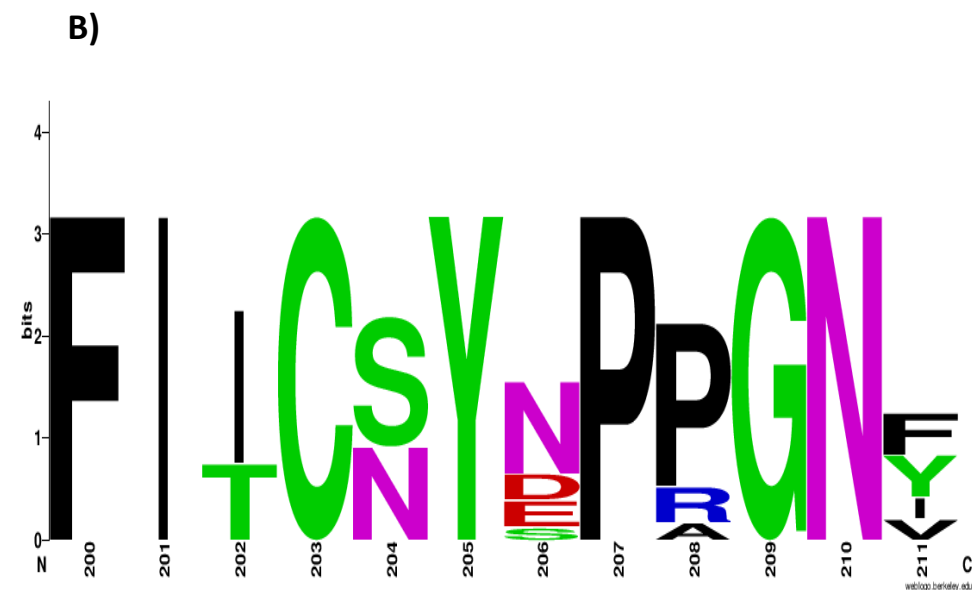

Supplement: Supplementary file 1 [file plants-12-01998-s001.zip › plants-2355924-supplementary.pdf]
